# Phytochemical analysis, antioxidant and anti-inflammatory potential of *FERETIA APODANTHERA* root bark extracts

**DOI:** 10.1186/s12906-017-2070-z

**Published:** 2018-01-12

**Authors:** Oluwayinka Olufunmilayo Owolabi, Dorcas Bolanle James, Ibrahim Sani, Binda T. Andongma, Opeoluwa O. Fasanya, Barnabas Kure

**Affiliations:** 1 Bioresources Development Centre, National Biotechnology Development Agency, Ya’adua way, Lugbe, Airport Road, Abuja, FCT Nigeria; 2 Biochemistry Department, Faculty of Science, Ahmadu Bello University, Zaria, Kaduna State Nigeria; 3 Petrochemical and Allied Department, National Research Institute for Chemical Technology, Zaria, Kaduna State Nigeria

**Keywords:** Phytochemical, Antioxidant, Inflammation, *Feretia apodanthera*, Extracts

## Abstract

**Background:**

Inflammation has been implicated in many disorders, including cancer and available therapies elicit adverse effects. Plants of the family Rubiaceae have shown potency against inflammation. The anti-inflammatory and anti-oxidant potential of *Feretia apodanthera* was investigated in this study to evaluate its effectiveness.

**Methods:**

The phytochemical, antioxidant and anti-inflammatory potential of root bark (n-Hexane, diethyl ether, ethanol and aqueous) extracts of *Feretia apodanthera* was investigated in this study. The extracts were subjected to various chemical tests for phytochemical constituents; their antioxidant activity was determined using in-vitro DPPH radical scavenging activity assay and their anti-inflammatory activity was determined using carrageenan induced paw oedema model. FTIR and GCMS analysis was done to determine the compounds present.

**Results:**

Phytochemical screening of extracts revealed the presence of unsaturated steroids, triterpenes, cardiac glycosides, tannins, saponin and alkaloids. Vitamin C had a median inhibitory concentration (IC_50_) of 0.038 mg/ml which was lower than IC_50_ of all the extracts. Of all the extracts, ethanol extract had the lowest IC_50_ (0.044 mg/ml) which is comparable to vitamin C. Anti-inflammatory studies showed that the inflammation inhibition potential of 400 mg/kg body weight of all the extracts was significantly lower (*p* < 0.05) than the standard ketoprofen (50 mg/kg) at the first three hours but significantly higher (*p* < 0.05) at the fourth hour. At the fifth hour, the inflammation inhibition potential of diethyl ether, ethanol and aqueous extracts were significantly higher (*p* < 0.05) than that of the standard. FTIR analysis showed the presence of ketones, amines, alkenes and carboxylic groups. GCMS analysis revealed compounds that are potential anti-inflammatory agents.

**Conclusion:**

This study revealed that extracts of *Feretia apodanthera* possess anti-inflammatory effects against right hind paw oedema of albino rats and can act as an effective antioxidant.

## Background

Plants have in one way or the other provided the basic need of mankind – food, shelter, clothing, protection from disease causing agents and treatment of various infections and illnesses since early days of human history [1]. Medicinal plants comprise of phytochemicals that improves the physiological balance of human beings and the knowledge of these healing properties has been passed down through generations. Traditional medicine is used by many African countries like Cameroon, Mali, Nigeria and Zambia to meet their health care needs [2, 3]. A vast range of medicinal plants around the globe has not yet been investigated to ascertain the claims made by traditional folks about their usefulness in treating diseases. Therefore, future research can be focused on medicinal plants.

Inflammation is usually a body response to tissue damage and to a number of systemic malfunctions including asthma, atherosclerosis, arthritis, physical injury and infection amongst many others [4]. Pro-inflammatory cytokines such as interleukin (IL)-1β, tumour necrosis factor (TNF)-α, and vascular endothelial growth factor (VEGF) mediate key points in inflammation [5]. Inflammation may be caused by foreign bodies, physical injury, allergens, radiation, stress, trauma, frostbite and alcohol amongst other things. Inflammation serves to isolate and eliminate infectious agents, induce repair and give protective response. Unfortunately, inflammation can lead to pain and discomfort that can persist for a long period of time [6]. Inflammation is manifested as pain, redness, loss of function of cells, swelling and heat. Based on its duration, inflammation is classified into acute and chronic inflammation.

*Feretia apodanthera* Del, is from the family Rubiaceae, a bush delicious shrub with winding or twisting branches, growing from 2 to 6 m tall [7], the leaf plate are elliptic to ovalate in shape with sometimes apiculate apex and rounded or cuneate base. The fruits are about 3-7 mm in diameter. It is commonly known as kuru-kuru by Hausa/Fulani - Northern Nigeria [8]. Traditionally, the root decortion of *F. apodanthera* is used to enhance erectile function of the penis and to treat stomach upset by the Hausa tribe of Kano State of Nigeria [8, 9].

Extracts from different part of the plant including the back, root and leaves have been used to treat renal and urinary infections, stomach ache, nausea, syphilis, infective wounds and other disease conditions [10, 11]. *Feretia apodanthera* is also used to enhance erectile function of the penis in some parts of Nigeria [8]. Mental conditions had been calmed using its extract. Taiwe et al. [12] reported that the extract of F. apodanthera was effective against cognitive errors, oxidative stress and seizures and in repairing memory impairment. Harman and tetrahydroharman isolated from methanol extract of *F. apodanthera* showed antimalarial activities and also a low cytotoxicity [13]. Recently, *Feretia apodanthera* extracts have also been shown to have high antioxidant activity similar to quercetin and relatively high flavonoid content [11]. The presence of these metabolites may be responsible for the therapeutic effect exhibited by this plant. Investigations with extract of *Feretia apodanthera* in rats also showed a decreased the activity of nuclear factor kappa β and nitric oxide which have been implicated in inflammation [14, 15].

The above mentioned factors have thus generated research interest in carrying out this study, thus this work is design to evaluate in-vitro antioxidant capacity and anti- inflammatory effect of different extracts of *Feretia apodanthera* against oedema in right hind paw of albino rats.

## Materials

### Plant material collection and identification

Fresh roots of *Feretia apodanthera Del* were gotten from its natural habitat at a local farm at Magami village, Gusau Local Government Area, Zamfara State, Nigeria in October, 2014. It was identified and authenticated at the Department of Biological Sciences (herbarium unit), Faculty of Science, Ahmadu Bello University, Zaria, Nigeria where a voucher number (930) was deposited for the specimen.

### Experimental animals

In this study, twenty five albino wistar rats of both sexes with weight ranging from 100 to 200 grammes were used. They were obtained from the National Institute for Trypanosomiasis Research animal house, Kaduna and housed based on the necessary environmental and nutritional conditions throughout the experiment. They were kept in polypropylene cages with paddy house bedding under standard laboratory condition for 14 days before the experiment was performed in order to acclimatize them. This study was permitted by Scientific and Biochemical Ethics Committee of the Faculty of Science, Ahmadu Bello University, Zaria, Kaduna State, Nigeria. The animals were provided with water ad libitum and laboratory chow. Best procedures were followed to minimize the animal used and reduce animal suffering.

## Method

### Preparation and extraction of plant material

The root bark was washed and then peeled with a clean knife by scraping. The scrapes were shade dried for two weeks at room temperature. The root back was further pulverized to powder form using a miller at Institute for Agricultural Research (IAR), Zaria. The powder was kept in a cool dry place for further use.

A modified method as described by James et al. [16] was used in this part of the study. The powdered sample (50 g each) was exhaustively extracted for 48 h by cold maceration extraction method using 500 ml of distilled water, ethanol, diethyl ether and n-hexane respectively. The mixture was passed through a mesh sieve (1 mm), filtered using Whatman filter paper no. 1, and then concentrated in a water bath at 45 °C. The extracts were kept in a deep freeze in a sample bottle awaiting use.

### Qualitative phytochemical screening of the extracts

Qualitative phytochemical screening was done on the four extracts of *Feretia apodanthera* using standard procedures described by Evans [17] to determine the phytochemicals present. The extracts (5 mg) were dissolved in 50 ml of the respective solvents used for their extraction. The solution was made ready for qualitative phytochemical analysis for carbohydrates, free reducing sugar, anthracene derivatives, cardiac glycosides, saponin, tannin, flavonoid, alkaloids, unsaturated sterols and triterpenes.

### In-vitro DPPH free radical scavenging antioxidant activity

The anti-oxidant activities of plant extracts was assessed using their ability to scavenge the activity of the free radicals of the stable 1,1-diphenyl-2-picrylhydrazyl (DPPH). The method of Chan et al. [18] was employed.

#### Principle

The 1,1-diphenyl-2-picrylhydrazyl (DPPH) is an oxidant having in its structure an odd electron. Its purple colour is reduced to yellow coloured diphenylpicrylhydrazine when it in contact with an antioxidant that can release a hydrogen atom or electron to it. The change in colour was measured at 520 nm using a UV/Visible light Spectrophotometer.

#### Procedure

DPPH solution was made by adding 6 mg of DPPH in 100 ml of methanol and allowing it to dissolve. About 2 ml of DPPH solution (0.1 mM) was added to 1 ml of various concentrations of the extracts (0.020, 0.040, 0.06, 0.080, 0.100 mg/ml). A mixture of methanol and DPPH was used a s control. All mixtures were vigorously shaken and made to stand in a dark place for 30 min. After this, solution absorbance was measured at 520 nm using a spectrophotometer. The experiments were performed in triplicates and the percentage scavenging activity of the extracts on DPPH radical was calculated on the basis of the formula below:

$$ \mathrm{Scavenging}\kern0.5em \mathrm{activity}=\frac{\left\{1\hbox{-} \mathrm{Absorbance}\kern0.5em \mathrm{of}\kern0.5em \mathrm{the}\kern0.5em \mathrm{sample}\right\}}{\mathrm{Absorbance}\kern0.5em \mathrm{of}\kern0.5em \mathrm{control}}\kern0.5em \times 100 $$

IC_50_ values were used to express the ability of the extracts to scavenge DPPH. The term “IC_50_” which connoted the concentration of the extract needed to scavenge 50% of DPPH radical, was calculated using the graph of scavenging activity plotted against sample concentration using Microsoft excel software.

### In-vivo anti-inflammatory activity determination of the extracts

A preliminary in-vivo anti-inflammatory test on carrageenan induced hind paw inflammation was carried out on the crude extracts to determine the most potent of them using a modified method of Kataki et al. [19]. The experimental animals was fasted for overnight prior to induction of edema, water was however available ad libitum.

Records of the weight of the rats were taken and the rats randomly separated into 5 cages representing 5 groups consisting of 3 rats each by matching rat with the highest weight with that of lowest weight and vice versa. The animals were fasted overnight prior to this investigation. Sub-plantar injection of 0.1 ml of 1% carrageenan in distilled water into the right hind paws of the animals was used to induce acute inflammation paw edema [20]. Water, ethanol, diethyl ether and *n-*hexane extracts were administered orally to each group just before carrageenan injection. Paw edema (expressed as an increase in paw volume) was measured using a digital caliper. Animals were deprived of water during this experimental period to ensure uniform hydration and reduce variability in edematous response. The animal grouping was as follows:

- **Group A:** Inflammation Control. Rats were induced with 0.1 ml of 1% carrageenan in distilled water. IC
- **Group B:** Rats were induced with 0.1 ml of 1% carrageenan and treated with 400 mg/kg of the n-hexane extract of *Feretia apodanthera.* IH
- **Group C:** Rats were induced with 0.1 ml of 1% carrageenan and treated with 400 mg/kg of the diethyl ether extract of *Feretia apodanthera.* ID
- **Group D:** Rats were induced with 0.1 ml of 1% carrageenan and treated with 400 mg/kg of the ethanol extract of *Feretia apodanthera.* IE
- **Group E:** Rats were induced with 0.1 ml of 1% carrageenan and treated with 400 mg/kg of the aqueous extract of *Feretia apodanthera.* IA
- **Group F:** Rats were induced with 0.1 ml of 1% carrageenan and treated with 50 mg/kg of the standard Ketoprofen*.* IS

The volume of right hind paw was measured at 1st, 2nd, 3rd, 4th and 5th hours after carrageenan injection. Increase in paw thickness and percent inhibition was calculated as below;

***Increse in Paw Volume = V***_***t***_ ***– V***_***0***_

Where;

V_t_ = Paw Volume at Time t.

V_0_ = Paw Volume at Time 0

% P***aw volume Inhibition = V***_***c***_***– V***_***t***_***/V***_***c***_***× 100***.

Where;

V_c_ = Paw Volume Increase in Control Animals.

V_0_ = Paw Volume Increase in Treated Animals.

Further analysis was carried out on the most potent fraction.

### Fourier transform infra-red spectroscopy analysis

The FTIR spectra of the fractions with the highest anti-inflammatory activity were carried out using FTIR-8400S spectrophotometer (Shidmazu model), available at the National Research Institute for Chemical Technology (NARICT) Laboratory Zaria. The paste from the ethanol and fractions were used in a form of a thin film, held in between two potassium bromide discs. The liquid paste was dropped on each disc and they spread into a thin film. The disc was then mounted in the FTIR spectrometer in the range 1.20 × 10^13^–1.20 × 10^14^ Hz) within electromagnetic spectrum of infrared section. Absorption is written in terms of wavenumbers (units cm^−1^).

### GCMS analysis of sample

Components of the sample were analyzed using a GC (Agilent 7890B) equipped with a HP-5 ms ultra inert column and coupled to a Mass spectrometer (Agilent 5977A). The sample was dissolved in methanol prior to analysis.

### Statistical analysis

The results were expressed as mean ± SD. Analysis of variance (ANOVA) was used to analyse data by the SPSS program (version 20.0 SPSS Inc. Chicago, IL, USA). Duncan Multiple Range Test was used to determine if the difference between the action of the different extracts across the animal groups were significant or not. *P* values less than 0.05 was considered significant (*p* < 0.05). The components were finally determined by matching the peaks’ mass spectra with that of the National Institute of Science and Technology (NIST) libraries mass spectral database at the Department of Analytical Chemistry, Ahmadu Bello University, Zaria.

## Result

### The percentage yield of extracts of Feretia apodanthera

The percentage yield of n-hexane, diethyl ether, ethanol and aqueous extracts of *Feretia apodanthera* is presented in Table 1. Aqueous extract had the highest yield (14.94%), ethanol extract had a yield of 9.20%, followed by diethyl ether extract (6.00%), while n-hexane extract has the lowest yield (4.54%). This showed that the percentage yield of crude *F. apodanthera* root bark extracts increased as polarity of the solvent of extraction used increased.

**Table 1:** Percentage Yield of n-Hexane, Diethyl Ether, Ethanol and Aqueous Extracts of *Feretia apodanthera* Root Bark

Extract	Amount recovered (g/50 g)	Percentage yield (%)
n-Hexane	2.72^a^	4.54
Diethyl ether	3.00^a^	6.00
Ethanol	4.60^b^	9.20
Aqueous	7.47^c^	14.94
S.E.M	1.09	
*P* value	0.05	

Values with different superscript down the column are significantly different (*p* < 0.05). *S.E.M* Standard error of mean

### The qualitative determination of phytochemicals in n-hexane, diethyl ether, ethanol and aqueous extracts of Feretia apodanthera

The qualitative determination of phytochemicals present in the n-hexane, diethyl ether, ethanol and aqueous extracts of *Feretia apodanthera* is presented in Table 2. Result showed carbohydrates, unsaturated sterols, reducing sugars, and triterpenes were present in all the four extracts. Tannins and flavonoids were present in diethyl ether, ethanol and aqueous extracts; reducing sugars and saponin were present in ethanol and aqueous extracts only. Ethanol extract contained a trace amount of alkaloids. Free anthraquinone were absent in all four extracts.

**Table 2:** The Qualitative Determination of Phytochemicals in n-Hexane, Diethyl Ether, Ethanol and Aqueous Extracts of *Feretia apodanthera*

Phytochemical	Method	N-hexane	Diethyl ether	Ethanol	Aqueous
Carbohydrates	Molish	–	–	+	+
Reducing Sugars	Fehling	–	–	+	+
Free Anthraquinone	Bontrager	–	–	–	–
Unsaturated steroids	Salkowski	+	+	+	+
Triterpenes	Liebermann-Bucchard	+	+	+	+
Cardiac glycosides	Keller-Killani	–	+	+	+
Saponin	Frothing	–	–	+	+
Tannins	Ferric chloride	–	+	+	+
Flavonoids	Shinoda	–	+	+	+
Alkaloids	Dragendoff	–	–	+	–

+ = Present, − = Absent

### The free radical scavenging ability of extracts on DPPH

A plot of percentage inhibition of free radicals versus extracts concentration depicts the antioxidant activity of the extracts of *Feretia apodanthera* based on their scavenging ability on DPPH which is a stable purple coloured radical and in the process reducing it into yellow coloured diphenylhydrazine. Antioxidant potential is inversely proportional to inhibitory concentration (IC_50_) value which was calculated from the linear regression of the percentage inhibition versus extract concentration.

**Fig. 1:**
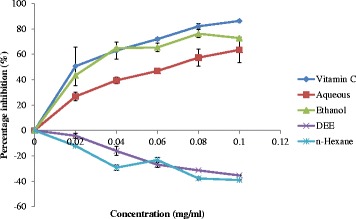
DPPH radical scavenging activities of n-hexane, diethyl ether, ethanol and aqueous extracts of *Feretia apodanthera* Discussion

The results of inhibition study are presented in Fig. 1 and Table 3. The results in Table 3 presents the 50% Inhibitory Concentration (IC_50_) values, the equation formular and DPPH radical scavenging activities at 1.00 mg/ml of n-hexane, diethyl ether, ethanol and aqueous extracts of *Feretia apodanthera*. The result shows that ethanol extract has the lowest IC_50_ value (0.053 mg/ml), followed by aqueous (0.063 mg/ml), then n-hexane extract (0.7499 mg/ml) and diethyl ether extract had the highest value of IC_50_ value (1.296 mg/ml) compared with vitamin C which had an IC_50_ of 0.048 mg/ml. The percentage free radical scavenging activity of the extract also showed that of all the extracts, ethanol extract had the highest percentage scavenging activity at all concentrations though it was significantly (*p* < 0.05) lower than the scavenging activity of vitamin C at the third, fourth and fifth hours respectively.

**Table 3:** DPPH Inhibitory Concentration (IC_50_) of the extracts of *Feretia apodanthera*

Extract	IC_50_ values (mg/ml)	Formula	Scavenging activity at 0.10 mg/ml (%)
n-Hexane	−0.131 (0.750) ^b^	y = −388.89× + 0.4762	−35.45 ± 0.46^a^(3.55 × 10^−36^)
Diethyl ether	−0.1127 (1.296) ^b^	y = −312.17× - 9.6296	−39.15 ± 0.46^a^(7.08 × 10^−40^)
Ethanol	0.053 ^a^	y = 350.53× + 43.466	72.75 ± 0.46^c^
Aqueous	0.064^a^	y = 457.67× + 19.312	63.49 ± 10.13^b^
Vitamin C	0.048^a^	y = 452.38× + 43.598	86.24 ± 0.46^d^
S.E.M	0.167		
*P*	0.05		

Values are written as means ± SD *n* = 3 replicate determinations values with different superscript down the column are significantly different (*p* < 0.05). Values with similar superscripts are not significantly different

### Anti-inflammation studies of Feretia Apodanthera extracts

The effect of the aqueous, ethanol, diethyl ether, n-Hexane extract of the root bark of *F. apodanthera* and the standard (ketoprofen) on carrageenan induced inflammation is presented in Table 4. The anti-inflammatory effects of the n-hexane, diethyl ether, ethanol and aqueous extracts of *F. apodanthera* was significantly (*p* > 0.05) lower than ketoprofen on carrageenan induced inflammation for the first, second and third hours. At the fourth and fifth hours, all the extracts exhibited a significantly (*p* < 0.05) higher anti-inflammatory potential than ketoprofen except for the n-hexane extract which showed no significant difference from the standard at the fifth hour only. Of all the extracts, ethanol extract had the highest inhibition of 93.35%.

**Table 4:** The Percentage Inhibition of the Aqueous, Ethanol, Diethyl Ether and n-Hexane Extract of the Root Bark of *Feretia Apodanthera* on Carrageenan Induced Inflammation

Extract	1st Hour (%)	2nd Hour (%)	3rd Hour (%)	4th Hour (%)	5th Hour (%)
n-Hexane (400 mg/kg)	12.47 ± 9.99^a^	24.11 ± 15.53^a^	18.21 ± 6.97^a^	77.83 ± 21.20^b^	30.28 ± 25.74^a^
Diethyl ether (400 mg/kg)	23.13 ± 7.15^a^	33.18 ± 9.00 ^a^	20.65 ± 13.11^a^	87.02 ± 8.83 ^b^	87.82 ± 8.21 ^b^
Ethanol (400 mg/kg)	16.85 ± 13.56^a^	33.65 ± 20.59^a^	30.43 ± 15.86^a^	89.73 ± 4.52 ^b^	93.35 ± 4.88 ^b^
Aqueous (400 mg/kg)	23.45 ± 11.18^a^	19.02 ± 10.14^a^	14.48 ± 14.13^a^	82.85 ± 11.93^b^	88.12 ± 7.80 ^b^
Standard (50 mg/kg)	45.90 ± 11.17^b^	69.94 ± 12.49^b^	72.55 ± 18.21^b^	45.83 ± 17.02^a^	48.23 ± 23.78^a^

Values are written as means ± SD n = 3 replicate determinations values with different superscript down the column are significantly different (p < 0.05). Values with similar superscripts are not significantly different

### FTIR spectra for ethanol extract of *F. apodanthera*

**Fig. 2:**
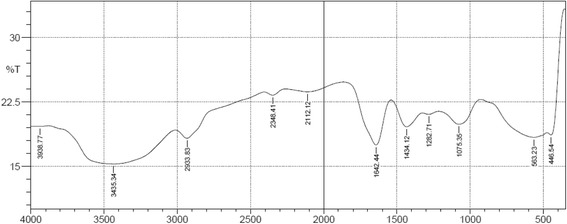
FTIR spectra for ethanol extract of *F. apodanthera*

Figure 2 shows the FTIR functional group spectra while Table 5 presents the summary of functional groups identified. Halide, alkyl, alkene, ketone, carboxylic acid were identified in the fraction.

**Table 5:** Functional Groups Identified by FTIR analysis of *F. apodanthera* Ethanol Extract

S/No	Absorption peak (cm^−1^)	Functional group	Assignment
1	446.54	R-I stretch	Alkyl halides
2	563.23	C-S stretch	Disulfides
3	1075.35	C-O-C	Cyclic ethers, large rings, C-O stretch
4	1282.71	P=O stretch	Organic nitrates or phosphates
5	1434.12	-CH_3_	Methyl bend
6	1642.44	C=C stretch	Alkenes
7	2112.12	C ≡ C stretch	Alkyne
8	2348.41	C=O	Ketones
9	2933.83	>CH_2_, C-H	Alkane
10	3435.34	R-C(O)-OH	Carboxylic acid
11	3938.77	N-H stretch	Amine

## Discussion

Many plant antioxidant potentials are related to their therapeutic potentials [21, 22]. The qualitative phytochemical screening of the extracts of *Feretia apodanthera* revealed the presence of saponins, triterpenes, tannins, flavonoids, cardiac glycosides and steroids which might be responsible for the obvious anti-inflammatory activities of the extracts of the plant. This is in line with the report of Ahmadiani et al.*,* [23] who stated that flavonoids as well as tannins possess anti-inflammatory effects. Flavonoids such as Quecetin are revealed to be useful in acute inflammation [24, 25]. Some flavonoids have a significant inhibitory potential against a wide array of enzymes such as phosphodiesterases, phospholipase A_2_, protein tyrosine kinases, protein kinase C, and others [26]. Manthey et al., [27] also reported that a number of flavonoids act by inhibiting key enzymes that are central in synthesizing prostaglandins processes. Triterpenoids may perform its anti-inflammatory action by reducing the cells that expresses inducible nitric acid synthase (iNOS), eg lupeol [28] or by inhibiting the production of nitric oxide by decreasing iNOS expression [29]. This shows that the high potential of these phytochemical compounds to inhibit inflammation processes is due to a decline in pro-inflammatory cytokines and iNOS production [30, 31]. Alkaloids is said to reduce the intensity of oedema caused by carrageenan by inhibiting vascular permeability induced by histamine [32].

The significant (*p* < 0.05) anti-inflammatory effect of the diethyl ether, aqueous and ethanol extracts may be due to the presence of flavonoids, triterpenes and tannins while the reduced anti-inflammatory effect of n-hexane is probably due to the absence of tannin, saponin and flavonoids. Ethanol extract showed the highest anti-inflammation potential, this may be due to a higher intensity of triterpenes and flavonoids, along with the presence of saponin, tannin, cardiac glycosides, steroids and alkaloids. This is in agreement with the work of Han and Bakovic [31] which suggests that triterpenoids are biologically active in producing anti-inflammatory effects and with the research done by Hamaleinen et al.*,* [33] that showed that flavonoids inhibits inflammation by inhibiting signal transducer and activator of transcription 1 (STAT-1) and nuclear factor kappa beta (NF-kβ) activations.

One of the primary methods for evaluating radical scavenging activity is by evaluating the ability of the antioxidants present in an extract to reduce purple coloured 1,2-diphenyl 2-picryl hydrazyl (DPPH] radical to yellow coloured diphenylpicrylhydrazine [31, 34]. A higher DPPH radical-scavenging activity is associated with a lower IC_50_ value [32]. Therefore, the ethanol extract had the highest DPPH reducing activity based on its relatively low IC_50_ values which was comparable with vitamin C as no significant (*p* < 0.05) difference was observed between their IC_50_ (Table 3). A positive result by the aqueous and ethanol extracts in this test indicates that they contain antioxidants that can scavenge free radicals [35]. Ethanol extract was followed by the aqueous extract, the n-hexane extract and the diethyl ether extract showed the least DPPH reducing activity due to its relatively high IC_50_ values. Previous studies on the DPPH radical scavenging of the aqueous and aqueous-acetone extract of *F. apodanthera* by Coulibaly et al. [11] showed lower IC_50_ values than all the extracts. This may be due to the difference in sample or extraction variation.

Carrageenan induced rat paw oedema is a well-established animal model for evaluating the anti-oedematous effect of drugs or compounds [36]. From the data presented on the anti-inflammation activity of the crude extracts, oedema was mildly inhibited by 400 mg/kg of the aqueous, ethanol, diethyl ether and n-hexane extracts from the first three hours (varying from 14.47 to 33.65%). This is in line with the report by Stark et al. [37] that showed that the root bark of several African plants from the family Rubiaceae possess anti-inflammatory and analgesic effects. When compared to the standard ketoprofen, a significantly (*p* < 0.05) higher inhibition of carrageenan induced inflammation was exhibited by the extracts in the fourth and fifth hours (77.83 to 93.35%) for all the extracts except for n-hexane which had a reduced inhibition of oedema in the fifth hour. This shows that while ketoprofen may act by inhibiting the secretion of serotonin, histamine and bradykinin, the extracts produce its anti-inflammatory effect in the second phase by limiting production of prostaglandin and cyclooxygenase (COX-2), making *Feretia apodanthera* a promising plant for further studies. This is in line with the studies that reveals that formation of oedema caused by carrageenan is in two phases; the first hour after carrageenan injection (first or early phase) involves the release of serotonin, histamine and bradykinin while the second or late phase (2–5 h) with increased oedema formation that remains up to the fifth hour involves the release of prostaglandins [38] COX-2 also induction in the hind paw [39].

For FTIR, the analysis time was less than five minutes and it required a minute quantity of the sample. The spectrum revealed the presence of functional groups such as aromatics, ethers, esters, alcohol, alkanes, ketones, alkenes, alkynes, amines and carboxylic acid [40]. Fink [41] in his report stated that the presence of keto carboxylate elicits antioxidant effect by scavenging hydrogen peroxide; compounds with aromatic rings and alcohol groups as seen in phenols have been known to modulate inflammation at different levels by decreasing the production of reactive nitrogen and oxygen species, limiting the activity of iNOS and COX, suppressing inflammatory chemokines and cytokines synthesis as well as controlling pathways for NF-κβ signaling [42].

GCMS analysis of *Feretia apodanthera* showed the presence of 2-Pentanone, 4-hydroxy-4-methyl-; Hexadecanoic acid, methyl ester; trans-9-Octadecenoic acid, pentyl ester; Methyl cyclohexanepropionate; Palmitoleic acid; 10-Undecenoyl chloride and Heptasiloxane, 1,1,3,3,5,5,7,7,9,9,11,11,13,13-tetradecamethyl- (Table 6). Hexadecanoic acid, methyl ester for example, is reported to reduce the production of nitric oxide- a key mediator in inflammation processes [35]- in cells by inhibiting arachidonic acid (Table 7) which is a precursor in the biosynthesis of prostagladins and therefore, inhibit the levels of pro-inflammatory mediators such as interleukin-10 (IL-10), prostaglandin (PGE_2_) and tumor necrosis factor-alpha (TNFα) [43]. Palmitoleic acid which was also identified in the extract was shown to have inflammatory lowering effects [44]. Heptasiloxane, 1,1,3,3,5,5,7,7,9,9,11,11,13,13-tetradecamethyl- have been identified in highly medicinal plants such as moringa leaves [45].

**Table 6:** GCMS Profiling of the Ethanol Extract of *Feretia apodanthera*

PK	RT	% Area	Compound name	CAS
1	5.4176	1.2033	2-Pentanone, 4-hydroxy-4-methyl-	000123–42-2
2	50.2953	0.1749	Hexadecanoic acid, methyl ester	000112–39-0
3	56.157	0.686	trans-9-Octadecenoic acid, pentyl ester	1,000,405–19-1
4	56.9995	0.109	Methyl cyclohexanepropionate	020681–51-0
5	60.9195	4.323	Palmitoleic acid	000373–49-9
6	62.3116	0.6729	10-Undecenoyl chloride	038460–95-6
7	90.3374	92.8309	Heptasiloxane, 1,1,3,3,5,5,7,7,9,9,11,11,13,13-tetradecamethyl-	019095–23-9

**Table 7:** Activity of Compounds Identified in the GCMS Study of Ethanol Extract of *Feretia apodanthera* Root Bark

S/No	Name of Compound	Molecular formula	Activity
1	2-Pentanone, 4-hydroxy-4-methyl-	C_6_H_12_O_2_	17-beta-hydroxysteroid dehydrogenase inhibitor, aryl-hydrocarbon-hydroxylase inducer, testosterone-hydroxylase-inducer, catechol-o-methyl-transferase inhibitor, methyl donor, methyl guanidine inhibitor
2	Hexadecanoic acid, methyl ester	C_17_H_34_O_2_	Acidulant, Acidifier, Arachidonic acid inhibitor, increase aromatic amino acid decarboxylase activity, inhibit production of uric acid, urinary-acidulant
3	trans-9-Octadecenoic acid, pentyl ester	C_23_H_44_O_2_	Arachidonic acid inhibitor, acidifier, acidulant, increase aromatic amino acid decarboxylase activity, catechol-o-methyl-transferase inhibitor, decrease glutamate oxaloacetate transaminase
4	Methyl cyclohexanepropionate	C_10_H_18_O_2_	catechol-o-methyl-transferase inhibitor, methyl donor, methyl-guanidine inhibitor
5	Palmitoleic acid	C_16_H_30_O_2_	Acidulant, Acidifier, Arachidonic acid inhibitor, increase aromatic amino acid decarboxylase activity, inhibit production of uric acid, urinary-acidulant
6	10-Undecenoyl chloride	C_11_H_19_ClO	–
7	Heptasiloxane, 1,1,3,3,5,5,7,7,9,9,11,11,13,13-tetradecamethyl-	C_14_H_44_O_6_Si_7_	

## Conclusion

These results suggest that the extract of *Feretia apodanthera* may possess some antioxidant properties and provide relief against inflammation making it a possible future therapy for inflammation. This may be the reason for the use of the extract in folklore medicine.

## Abbreviations

COX-2: Cyclooxygenase 2
DPPH: 1,1-diphenyl-2-picrylhydrazyl
*F. apodanthera*: *Feretia apodanthera*
FTIR: Fourier transform infrared
GCMS: Gas chromatography mass spectrometry
IAR: Institute for Agricultural Research
IC_50_: 50% Inhibitory concentration
IL: Interleukin
iNOS: Inducible nitric acid synthase
NF-kβ: Nuclear factor kappa beta
STAT 1: Signal transducer and activator of transcription 1
TNF α: Tumour necrosis factor alpha
VEGF: Vascular endothelial growth factor
